# Association of work-related musculoskeletal disorders and work-related factors with quality of life among university teachers in Taiwan: a cross-sectional study

**DOI:** 10.1186/s12889-026-28731-0

**Published:** 2026-08-05

**Authors:** Chih-Ching Liu, Yu-Hsuan Hung, Chien-Hui Liu, Kai-Ren Chen, Jiun-Yi Wang

**Affiliations:** 1 Department of Health Care Management, National Taipei University of Nursing and Health Sciences, No. 365, Ming-te Rd., Peitou Dist., Taipei City, 112303 Taiwan; 2 Department of Healthcare Administration, College of Medical and Health Science, Asia University, No. 500, Lioufeng Rd., Wufeng, Taichung City, 413305 Taiwan; 3 Division of Emergency Medical Service, New Taipei City Fire Department, New Taipei City, Taiwan; 4 Department of Public Health, Fu Jen Catholic University, New Taipei City, Taiwan; 5 Department of Medical Research, China Medical University Hospital, China Medical University, Taichung City, Taiwan

**Keywords:** Work-related musculoskeletal disorders, Work-related factors, Quality of life, University teachers

## Abstract

**Background:**

Work-related musculoskeletal disorders (WMSDs) are common and distressing issues among university teachers, and various occupational factors may affect their quality of life (QoL). However, empirical evidence examining the association between QoL, WMSDs, and work-related factors remains limited. This study aimed to investigate the overall and domain-specific QoL among university teachers with WMSDs in Taiwan and its association with the number of body regions affected by WMSDs and work-related factors.

**Methods:**

A cross-sectional survey was conducted to reach full-time university teachers with WMSDs. Data were collected through a self-administered digital questionnaire that incorporated the Nordic Musculoskeletal Questionnaire, work-related factors, and the World Health Organization Quality of Life–BREF. Multivariable linear regression models were applied to examine the associations of overall and domain-specific QoL scores with the number of body regions affected by WMSDs and work-related factors.

**Results:**

A total of 262 participants were enrolled. The mean overall QoL score was 57.17 ± 8.80, with the psychological domain showing the lowest mean score among the four QoL domains. A greater number of WMSD-affected body regions was significantly associated with lower overall QoL (β=-0.722, *p* = 0.001) and poorer scores across all QoL domains. Having three or more days off per week was significantly associated with higher physical (1.43, *p* = 0.009), psychological (1.57, *p* = 0.016), and environmental (1.73, *p* = 0.003) QoL, while being an associate professor (-0.93, *p* = 0.036) was related to lower social QoL.

**Conclusion:**

Significant associations were observed between both overall and domain-specific QoL scores and the number of body regions affected by WMSDs, as well as specific work-related characteristics. The findings suggest that interventions targeting musculoskeletal health, sufficient recovery time, and psychosocial support may help improve the well-being of university teachers. Longitudinal studies are warranted to confirm these associations.

## Introduction

Work-related musculoskeletal disorders (WMSDs) have been identified by the International Labour Organization as the one of the most common occupational disease worldwide [1]. WMSDs are characterized by pain or functional impairment in muscles, tendons, or joints, typically resulting from repetitive tasks, prolonged postures, or insufficient rest [2]; such conditions are also commonly observed in the work lives of university teachers [3–5]. Previous studies have reported that the prevalence of self-reported WMSDs among university teachers ranges from approximately 55% to 88%, often affecting multiple anatomical regions [3–5], underscoring the substantial burden of WMSDs in this population.

WMSDs not only result in physical impairment but also impose indirect burdens, including reduced work productivity, an increased risk of secondary injuries, socioeconomic strain, and a diminished ability to participate in social activities and maintain relationships with friends and family [6]. Consequently, they may disrupt multiple domains of an individual’s life—physical, psychological, social, and environmental—and contribute to declines in overall QoL [7, 8]. Previous studies have found that university teachers tend to report lower QoL compared to the general population [9]. Therefore, a better understanding of QoL profiles and associated factors among university teachers affected by WMSDs is essential to inform healthcare professionals in the development and implementation of effective strategies to enhance the QoL of this population.

Over the past decades, growing attention has focused on the association between musculoskeletal disorders (MSDs) and QoL among school teachers [7, 10–13]. Evidence indicates that the presence of MSDs [7, 12] —particularly when multiple anatomical sites are affected [10, 11, 13] —is significantly associated with poorer overall, as well as physical and mental QoL, as measured by the SF-36 or SF-12. However, to the best of our knowledge, among them, only one study has specifically investigated the association between WMSDs and QoL [7]. This study relied on dichotomized measures of WMSDs (yes/no) and used t-tests to compare QoL between school teachers with and without WMSDs, without evaluating the associations of the number of WMSD sites or work-related factors with QoL using multivariable regression analyses [7]. Moreover, this study has not examined the social and environmental dimensions of QoL, which can be assessed using the World Health Organization Quality of Life-BREF (WHOQOL-BREF) [14]. Furthermore, this study only focused on the teachers in the kindergarten, primary, preparatory and secondary schools. The results may be not generalized to the university teachers [7].

Although university teachers face similar pressures to those reported by educators at other levels of education [15], previous studies have identified several working conditions as stressors unique to academic careers [16, 17]. These stressors arise from working conditions unique to university teachers, including a strong reliance on research publications not only to enhance individual academic reputation and financial security, but also to improve the ranking, funding, and institutional prestige of their employing institutions [16, 17]. In addition to facing continuous pressure to achieve academic qualifications and advance scientifically, they frequently engage with heterogeneous student populations, manage high student turnover, undertake substantial teaching workloads, and fulfill multiple institutional responsibilities [18–20]. These high workload demands may require them to stand for prolonged periods while teaching in class [3] or spend extended hours sitting in their offices, often surrounded by computers [3, 4], while maintaining static postures during task performance [3]. Additionally, these high workload demands may also prevent university teachers from having adequate breaks for resource replenishment and recovery, ultimately resulting in increased difficulty recovering from stress, and ultimately, diminishes their QoL [21]. Moreover, university teachers at different job ranks may perceive and manage their workload differently, which may lead to differences in QoL across job ranks. Compared with professors, assistant and associate professors may experience greater work-related stress due to concerns about limited career advancement, which is often contingent upon their research productivity [22]. Additionally, compared with assistant professors, associate professors are often expected to undertake more teaching and research responsibilities with a higher degree of work autonomy [23], which may increase much more stress during their work. In contrast, although full professors are often expected to serve as pace-setters in scientific research and to undertake greater teaching and research responsibilities [22], these demands may not necessarily result in higher levels of stress, as they may derive a stronger sense of professional identity from their work [24] and benefit from broader social support networks [9].

Given that the aforementioned work-related factors—including job rank, working hours, teaching hours, number of days off, opportunities for rest breaks at work, and administrative responsibilities—may be associated with overall and domain-specific QoL, it is essential to jointly examine these factors alongside work-related musculoskeletal factors within a unified multivariable framework to obtain a more comprehensive understanding of the factors associated with overall and domain-specific QoL among university teachers with WMSDs. However, although a number of studies have examined overall [24–26] and domain-specific QoL [24–31] among university teachers, reporting mean overall QoL scores ranging from 58.32 to 70.75 [24–26], with relatively lower scores in the psychological [25, 28–30] and environmental [24–27, 31] domains, to the best of our knowledge, only two studies have examined the associations between work-related factors and specific QoL domains using multivariable analyses among university teachers. The findings of these few studies were inconsistent and restricted to the physical and mental domains of QoL, as assessed by the SF-36 [9, 32]. For the physical QoL domain, one study found that daily working hours and job rank were significantly associated factors [9], whereas another study reported no significant associations [32]. For the mental QoL domain, one study found no association with daily working hours and job rank [9], while another study did not include these factors in the analysis [32]. Additionally, one study reported a significant association between the number of SCI papers and QoL [32], whereas another study found no significant relationship [9]. These inconclusive findings may be attributable to the omission of other potential work-related factors—such as number of days off, opportunities for rest breaks at work, and administrative responsibilities—in the multivariable regression analyses. Additionally, these studies did not take into account the number of affected anatomical sites and focus on university teachers with WMSDs [9, 32].

To address the aforementioned research gap, the present study investigated the profiles of QoL and the associations of specific work-related factors—including academic rank, working hours, teaching hours, number of days off, opportunities for rest breaks at work, and administrative responsibilities—with overall and domain-specific QoL among university teachers with WMSDs. These Findings from the study can provide evidence for developing occupational health strategies to improve the well-being and sustainability of the academic workforce.

## Methods

### Participants and data source

Data for this cross-sectional study were obtained from a previous survey on the prevalence of WMSDs among university teachers in Taiwan. Details of the sampling design are provided elsewhere [4]. In brief, full-time university teachers with a PhD degree and holding the rank of assistant, associate, or full professor were recruited, while participants with non-work-related musculoskeletal disorders were excluded. The required sample size was originally calculated to be 295 based on the setting regarding the precision of estimated prevalence in a previous study [4]. A convenience sampling was conducted from universities across four geographic regions between April and May 2022. An invitation email containing a link to an anonymous, self-administered online questionnaire was sent to eligible participants from 40 out of 126 universities in Taiwan, while the 40 universities were randomly selected across five regions of Taiwan to enhance geographic diversity. The questionnaire’s introductory page described the study objectives, obtained informed consent, and explained the voluntary nature of participation. Because the researchers closed the link of google form when responses received exceeded the required number, the ratio of responses received to the total emails sent to university teachers might not reflect the true response rate. Therefore, response and non-response rates were not reported.

Based on the observed sample size (262 participants with WMSDs) and a fair effect size of 0.25 in a linear multiple regression model (Fixed effect, R-square deviation from zero), the estimated posteriori power was 99% by using G-power calculator.

### Assessment of QoL

QoL was assessed using the Taiwanese version of the WHOQOL-BREF [33], which is a self-administered tool comprising 26 items: two assessing overall QoL and general health, and 24 items across four domains—Physical Health (7 items: pain, energy, sleep, mobility, daily activities, dependence on treatment, work capacity), Psychological Health (6 items: positive/negative feelings, cognition, self-esteem, body image, spirituality), Social Relationships (3 items: personal relationships, social support, sexual activity), and Environment (8 items: safety, home, finances, health/social care, learning opportunities, recreation, physical environment, transportation) [14].

The Taiwanese WHOQOL-BREF, comprising 26 items plus two culturally relevant items (‘being respected’ in Social Relationships and ‘food availability’ in Environment), was used to assess QoL among university teachers with WMSDs [14, 34]. Items were rated on a five-point Likert scale, with domain scores (range 4–20) calculated per the user manual; higher scores indicate better QoL. The Chinese version is widely validated [34]. A pretest with 30 participants showed acceptable internal consistency: Physical Health α = 0.815, Psychological α = 0.860, Social Relationships α = 0.710, Environment α = 0.840, overall α = 0.90.

### Exposure variables: assessment of WMSDs and work-related factors

WMSDs were identified using the Nordic Musculoskeletal Questionnaire (NMQ) [35, 36], a widely used instrument for identifying self-reported musculoskeletal symptoms [4, 5]. The NMQ evaluates discomfort in nine anatomical regions: the neck, shoulders, wrists/hands, elbows, upper back, waist/lower back, hips/thighs, knees, and ankles/feet. Participants were asked to report the presence of pain or functional impairment in muscles, tendons, or joints in these nine regions.

Participants reporting work-related discomfort in at least one anatomical region were classified as having WMSDs and were eligible for inclusion in the present analysis. The number of WMSD-affected body regions was calculated by summing the affected anatomical regions, yielding a possible range of 1–9.

In addition to WMSD assessment, information on work-related factors, including academic title, average days off per week, working hours per day, teaching hours per day, breaks to rest at work, and administrative positions, was collected using a structured questionnaire developed by the research team. These work-related details were based on conditions within the past academic year.

### Confounding variables

Information on sociodemographic characteristics and health-related factors potentially associated with QoL [9, 28, 32, 37–39] was also collected using a structured questionnaire. Sociodemographic variables included sex (men or women), age (in years), and marital status (single vs. married). Health-related variables were assessed based on conditions during the previous academic year, including regular exercise habits (yes vs. no), average sleeping hours per day, participation in health promotion programs (yes vs. no), body weight, height, and history of chronic diseases (yes vs. no). Regular exercise habits were defined based on participation in moderate-to-vigorous physical activity. Body mass index (BMI) was calculated as weight in kilograms divided by height in meters squared (kg/m²) and categorized into three groups: underweight/normal (< 24.0 kg/m²), overweight (24.0–26.9 kg/m²), and obese (≥ 27.0 kg/m²) [40]. Average daily sleep duration was categorized into two groups: <6 h per night and ≥ 6 h per night [41]. Participants with a history of chronic illness were asked to report specific conditions, including diabetes, hypertension, peptic ulcer disease, thyroid disease, and other chronic diseases.

### Statistical analysis

We first note that the collected dataset did not have any missing data because a reminder would appear once participants had missed any items in the online survey. Descriptive statistics were used to summarize participants’ sociodemographic, health, and work-related factors, with categorical variables reported as frequencies (%) and continuous variables as means ± standard deviation (SD). Means ± SD of QoL facet and domain scores were reported. Given that previous studies have commonly categorized the number of musculoskeletal disorder sites among teachers using the median number of affected regions [10, 11, 13], the number of WMSD-affected body regions was dichotomized at the median (< median vs. ≥ median) in the present study to facilitate comparison with existing literature and provide an interpretable classification of WMSD burden. Group comparisons were performed using independent t-tests and one-way analysis of variance (ANOVA), as appropriate. Multivariable linear regression was used to examine the associations of WMSD burden and work-related factors with overall QoL as well as its four domains. In the primary analysis, the number of WMSD-affected body regions was entered as a dichotomous variable (< median vs. ≥ median). Regression coefficients (β) and 95% confidence intervals (CIs) were reported. All models were adjusted for sociodemographic and health-related variables considered potential confounders based on previous literature [4, 9, 28, 32, 37–39]. All confounders included in the regression analyses were treated as categorical variables and entered into the regression models using k − 1 dummy coding.

Because categorization of a continuous variable may result in loss of information, additional regression analyses were conducted treating the number of WMSD-affected body regions as a continuous predictor to examine the dose–response association with overall QoL and its individual domains.

The assumptions of linear regression were assessed. The linearity and homoscedasticity assumptions were evaluated through graphical inspection of residuals versus predicted values, which showed randomly distributed residuals around zero without apparent systematic patterns. The normality of residuals was examined using Q–Q plots, and no substantial violations of regression assumptions were observed. Moreover, the Durbin–Watson statistics were close to 2, indicating that the residuals of the multiple regression models were independent and that no significant autocorrelation was present. Multicollinearity was assessed using VIFs (< 3.0 for all variables). Analyses were conducted with SAS 9.4 (SAS Institute Inc., Cary, NC), with *p* < 0.05 considered significant.

## Results

### Characteristics of the study participants

Among 262 participants with WMSDs, they were recruited from 24 universities (northern: 9, central: 10, southern: 3, eastern: 2), with 52.2% employed in public institutions. Their mean teaching tenure was 16.87 ± 10.63 years, and academic fields included humanities (24.05%), engineering (23.28%), medicine (14.89%), management (17.94%), and other disciplines (19.85%). Additionally, most participants were men (60.3%), married (83.2%), exercised regularly (69.9%), and slept ≥ 6 h/day (81.3%), with a mean age of 50.6 ± 7.9 years. Only 22.9% had attended health promotion courses, while 48.1% reported having chronic diseases, and the mean BMI was 24.7 ± 3.6 kg/m². WMSDs most commonly affected the shoulders (85.5%), neck (82.4%), and waist/ lower back (76.0%), with a median of four body regions affected. Regarding work, 30.9% were professors, 59.5% reported only one day off per week, 69.9% held no administrative positions, and 40.8% took rest breaks (Table 1).

**Table 1:** Characteristics of study participants (*N* = 262)

Variables	*n*	%
**Sociodemographic and health characteristics**		
Sex		
Men	158	60.31
Women	104	39.69
Age (years)		
< 45	69	26.33
45–54	94	35.88
≥ 55	99	37.79
Mean ± SD	50.59 ± 7.93	
Marital status ^a^		
Single	43	16.80
Married	213	83.20
Regular exercise habits ^b^	183	69.85
Average sleeping hours per day ^b^		
< 6	49	18.70
≥ 6	213	81.30
Participating any health promotion courses ^b^	60	22.90
Body Mass Index (BMI)		
≤ 23	125	47.71
24–26	77	29.39
≥ 27	60	22.90
Mean ± SD	24.67 ± 3.57	
History of chronic disease	126	48.09
WMSDs in specific body region		
Neck	216	82.44
Shoulders	224	85.50
Wrists/ Hands	152	58.02
Elbow	131	50.00
Upper back	177	67.56
Waist/ Lower back	199	75.95
Hips/ Buttocks	118	45.04
Knees	146	55.73
Ankles/ Feet	123	46.95
Number of body regions affected by WMSDs		
< Median ^c^	92	35.11
≥ Median	170	64.89
**Work-related characteristics**		
Academic title		
Assistant professor	102	38.93
Associate professor	79	30.15
Professor	81	30.92
Average days off per week ^d^		
1	156	59.54
2	87	33.21
≥ 3	19	7.25
Average working hours per day ^d^		
≤ 8	78	29.77
9–10	74	28.24
≥ 11	110	41.99
Average teaching hours per week ^d^		
≤ 8	54	20.61
9–10	101	38.55
≥ 11	107	40.84
Breaks to rest at work ^d^		
Yes	107	40.84
No	155	59.16
Administrative positions ^d^		
Yes	79	30.15
No	183	69.85
Total	262	100.00

*WMSDs *work-related musculoskeletal discomforts

^a^Inconsistency between total population and population summed for individual variables was due to missing information

^b^Within the past year

^c^Median = 4

^d^Within the past year

## Facet and domain scores of the QoL related to WMSDs

Table 2 presents QoL facet and domain scores overall and by the number of body regions affected by WMSDs. The mean total QoL score was 57.17 ± 8.80, with the lowest scores for positive feelings (Q5) and general health (Q2), and the highest for dependence on medication (Q4) and opportunities for new information/skills (Q13). Domain means were 14.46 ± 2.47 (physical), 14.01 ± 2.57 (psychological), 14.11 ± 2.62 (social), and 14.59 ± 2.33 (environment), with the psychological domain lowest and environment highest. Participants with < median affected regions had significantly higher total and domain-specific QoL scores than those with ≥ median regions.

**Table 2:** Facet and domain scores of the QoL by the number of body regions affected by WMSDs (*N* = 262)

Facets or Domains of QoL	Overall		Number of body regions affected by WMSDs				*p* ^b^
			< Median ^a^		≥ Median		
	Mean	SD	Mean	SD	Mean	SD	
Total^c^	57.17	8.80	59.64	8.07	55.82	8.90	0.001*
Q1 Overall Quality of Life	3.23	0.80	3.50	0.72	3.08	0.80	< 0.001*
Q2 General health	2.98	0.85	3.21	0.82	2.85	0.84	0.001*
**Physical domain** ^**d**^	14.46	2.47	15.47	2.06	13.92	2.51	< 0.001*
Q3 Pain and discomfort ^e^	3.83	0.85	4.09	0.71	3.69	0.88	< 0.001*
Q4 Dependence on medication or treatment ^e^	4.16	0.90	4.45	0.76	4.01	0.94	< 0.001*
Q10 Energy and fatigue	3.08	0.84	3.34	0.76	2.94	0.85	< 0.001*
Q15 Mobility	3.89	0.85	4.17	0.72	3.74	0.87	< 0.001*
Q16 Sleep and rest	3.02	1.01	3.34	0.92	2.85	1.02	< 0.001*
Q17 Daily activities	3.62	0.83	3.92	0.71	3.46	0.84	< 0.001*
Q18 Work capacity	3.69	0.82	3.76	0.87	3.66	0.79	0.337
**Psychological domain** ^**d**^	14.01	2.57	14.52	2.48	13.74	2.59	0.018*
Q5 Positive feelings	2.94	0.91	3.15	0.96	2.83	0.86	0.006*
Q6 Spirituality	3.84	0.92	3.95	0.93	3.78	0.91	0.155
Q7 Thinking, learning, memory, and concentration	3.52	0.92	3.71	0.87	3.42	0.93	0.015*
Q11 Bodily image and appearance	3.60	0.89	3.71	0.85	3.54	0.90	0.136
Q19 Self-esteem	3.73	0.78	3.79	0.82	3.70	0.75	0.353
Q26 Negative feelings ^e^	3.39	0.82	3.48	0.83	3.35	0.81	0.216
**Social relationships domain** ^**d**^	14.11	2.62	14.55	2.56	13.86	2.64	0.042*
Q20 Personal relationships	3.66	0.78	3.76	0.73	3.61	0.80	0.140
Q21 Sexual activities	3.31	0.96	3.37	0.98	3.27	0.95	0.426
Q22 Social support	3.70	0.75	3.84	0.65	3.62	0.79	0.002*
Q27 Being respected ^f^	3.44	0.81	3.59	0.79	3.36	0.81	0.029*
**Environment domain** ^**d**^	14.59	2.33	15.10	2.04	14.31	2.43	0.005*
Q8 Physical security and protection	3.63	0.87	3.82	0.86	3.54	0.86	0.013*
Q9 Physical environment (pollution/ noise/ traffic/ climate)	3.16	0.95	3.38	0.82	3.04	0.99	0.004*
Q12 Financial resources	3.47	0.93	3.57	0.93	3.41	0.93	0.204
Q13 Opportunities for acquiring new information and skills	3.99	0.73	4.13	0.63	3.92	0.77	0.017*
Q14 Participation in and opportunities for recreation/ leisure activities	3.12	1.00	3.16	0.92	3.09	1.04	0.596
Q23 Home environment	3.77	0.76	3.87	0.73	3.72	0.77	0.122
Q24 Health and social care: accessibility and quality	3.77	0.78	3.91	0.69	3.69	0.81	0.025*
Q25 Transportation	3.94	0.74	4.09	0.69	3.86	0.75	0.020*
Q28 Food availability ^f^	3.97	0.71	4.05	0.72	3.92	0.71	0.139

*WMSDs *work-related musculoskeletal discomforts, *SD* standard deviation

^a^Median = 4

^b^Based on independent samples t test

^c^The total quality of life score was calculated by summing the scores of four domains: Physical, Psychological, Social relationships, and Environmental domains

^d^The scores of items within the same domain were summed, averaged, and then multiplied by four, resulting in a total score ranging from 4 to 20

^e^Q3, Q4, and Q26 are reverse-coded questions

^f^The national item of WHOQOL-BREF Taiwan version Q27 ‘being respected’ is included in the social relationships domain (TW), and Q28 ‘food availability’ is included in the environment domain (TW)

### Association between WMSDs, work-related factors, and QoL

Table 3 and 4 present the associations between the number of body regions affected by WMSDs, work-related factors, and QoL, with Tables 3 reporting overall QoL and Table 4 reporting QoL across the four specific domains.

**Table 3:** Associations of the overall QoL with the number of body regions affected by WMSDs and work-related factors among university teachers (*N* = 262)

Variables	QoL		*p*	Crude β	95%CI	*P*	Adjusted β^d^	95%CI	*p*
	Mean	SD							
Number of body regions affected by WMSDs			0.001^a^ *						
< Median ^e^	59.64	8.07		0			0		
≥ Median	55.82	8.90		-3.80*	-6.06~-1.54	0.001	-3.11*	-5.35~-0.87	0.007
				Trend test: ß= -0.989, *p* < 0.001			Trend test: ß= -0.722, *p* = 0.001		
**Work-related characteristics**									
Academic title			0.361 ^b^						
Assistant professor	56.74	9.69		0			0		
Associate professor	56.53	8.43		-0.14	-2.82 ~ 2.54	0.920	-1.75	-4.46 ~ 0.97	0.206
Professor	58.32	7.91		1.80	-0.82 ~ 4.42	0.178	-1.53	-4.51 ~ 1.45	0.314
Average days off per week ^f^			0.002 ^b, c^*						
1	55.95	9.24		0			0		
2	58.10	7.45		2.24	-0.11 ~ 4.59	0.061	1.75	-0.64 ~ 4.13	0.150
≥3	62.86	8.40		6.26*	2.01 ~ 10.52	0.004	5.64*	1.45 ~ 9.83	0.009
Working hours per day ^f^			0.356 ^b^						
≤ 8	58.29	9.45		0			0		
9–10	57.08	9.39		-0.92	-3.83 ~ 1.98	0.531	1.04	-1.72 ~ 3.80	0.460
≥ 11	56.42	7.85		-1.63	-4.28 ~ 1.01	0.225	0.98	-1.67 ~ 3.64	0.466
Teaching hours per week ^f^			0.403 ^b^						
≤ 8	58.53	7.06		0			0		
9–10	57.09	9.11		-1.49	-4.47 ~ 1.50	0.327	-1.11	-4.08 ~ 1.85	0.460
≥ 11	56.55	9.27		-2.06	-5.02 ~ 0.89	0.170	-1.66	-4.71 ~ 1.39	0.285
Breaks to rest at work ^f^			0.253 ^a^						
No	56.65	8.80		0			0		
Yes	57.91	8.77		1.20	-1.04 ~ 3.44	0.292	0.50	-1.66 ~ 2.65	0.649
Administrative positions ^f^			0.233 ^a^						
No	56.78	9.45		0			0		
Yes	58.05	7.02		1.46	-0.94 ~ 3.86	0.234	1.16	-1.22 ~ 3.54	0.337

*WMSDs *work-related musculoskeletal discomforts, *CI *confidence interval, *β* regression coefficient

^a^Based on independent samples t test

^b^Based on one-way ANOVA

^c^Significant differences between an average of 1 and ≥ 3 days off per week in Scheffé’s post-hoc test

^d^Estimated from the multivariable linear regression model with adjustment for sex, age, marital status, regular exercise habits, sleeping hours, participating any health promotion courses, BMI, and history of chronic disease

^e^Median = 4

^f^Within the past year

* *P*<0.05

**Table 4:** Associations of the four QoL domains with the number of body regions affected by WMSDs and work-related factors among university teachers (*N* = 262)

Variables	Physical			Psychological			Social relationships			Environment		
	Adjusted β^a^	95% CI	*p*	Adjusted β^a^	95% CI	*p*	Adjusted β^a^	95% CI	*p*	Adjustedβ^a^	95% CI	*p*
Number of body regions affected by WMSDs												
<Median^b^	0		< 0.001	0		0.107	0		0.082	0		0.016
≥ Median	-1.17*	-1.75~ -0.60		-0.56	-1.24 ~ 0.12		-0.63	-1.35 ~ 0.08		-0.74*	-1.35~ -0.14	
	Trend test: ß=-0.262, *p* < 0.001			Trend test: ß= -0.153, *p* = 0.022			Trend test: ß= -0.148, *p* = 0.035			Trend test: ß=-0.159, *p* = 0.007		
**Work-related characteristics**												
Academic title												
Assistant professor	0			0			0			0		
Associate professor	-0.47	-1.17 ~ 0.22	0.179	-0.19	-1.02 ~ 0.63	0.646	-0.93*	-1.80~ -0.06	0.036	-0.15	-0.88 ~ 0.58	0.685
Professor	-0.22	-0.99 ~ 0.54	0.563	-0.50	-1.40 ~ 0.41	0.282	-0.42	-1.38 ~ 0.53	0.381	-0.38	-1.19 ~ 0.42	0.350
Average days off per week ^c^												
1	0			0			0			0		
2	0.55	-0.06 ~ 1.15	0.079	0.26	-0.47 ~ 0.98	0.484	0.06	-0.70 ~ 0.82	0.875	0.88*	0.24 ~ 1.53	0.007
≥ 3	1.43*	0.36 ~ 2.50	0.009	1.57*	0.29 ~ 2.84	0.016	0.92	-0.42 ~ 2.26	0.176	1.73*	0.60 ~ 2.86	0.003
Working hours per day ^c^												
≤ 8	0			0			0			0		
9–10	0.26	-0.44 ~ 0.97	0.467	0.39	-0.45 ~ 1.23	0.361	0.30	-0.58 ~ 1.18	0.499	0.08	-0.66 ~ 0.83	0.824
≥ 11	0.22	-0.46 ~ 0.90	0.517	0.19	-0.62 ~ 1.00	0.642	0.42	-0.42 ~ 1.27	0.326	0.15	-0.57 ~ 0.86	0.689
Teaching hours per week ^c^												
≤ 8	0			0			0			0		
9–10	-0.41	-1.17 ~ 0.34	0.284	-0.42	-1.32 ~ 0.48	0.359	-0.17	-1.12 ~ 0.78	0.724	-0.11	-0.91 ~ 0.69	0.787
≥ 11	-0.52	-1.30 ~ 0.26	0.190	-0.60	-1.53 ~ 0.33	0.205	-0.15	-1.12 ~ 0.82	0.762	-0.39	-1.22 ~ 0.43	0.347
Breaks to rest at work ^c^												
No	0			0			0			0		
Yes	-0.04	-0.59 ~ 0.51	0.897	0.24	-0.41 ~ 0.90	0.465	0.09	-0.60 ~ 0.77	0.806	0.21	-0.38 ~ 0.79	0.486
Administrative positions ^c^												
No	0			0			0			0		
Yes	0.20	-0.41 ~ 0.80	0.528	0.40	-0.33 ~ 1.12	0.281	0.32	-0.44 ~ 1.08	0.409	0.25	-0.39 ~ 0.89	0.442

*WMSDs *work-related musculoskeletal discomforts, *CI *confidence interval, *β *regression coefficient

^a^Estimated from the multivariable linear regression model with adjustment for sex, age, marital status, regular exercise habits, sleeping hours, participating any health promotion courses, BMI, and history of chronic disease

^b^Median = 4

^c^Within the past year

* *P*<0.05

Table 3 shows significant differences in mean QoL scores by number of WMSD-affected regions (*p* = 0.001) and days off per week (*p* = 0.002). Participants with < median affected regions reported higher QoL (59.64 ± 8.07) than those with ≥ median regions (55.82 ± 8.90). QoL was highest among those with three days off per week (62.86 ± 8.40), followed by two (58.10 ± 7.45) and one day (55.95 ± 9.24).

In the multivariable linear regression analysis, participants with a median or greater number of body regions affected by WMSDs had significantly lower QoL scores compared to those with fewer than the median number of affected regions (β = -3.11; 95% CI, -5.35 to -0.87). Additionally, the trend analysis revealed a significant inverse gradient association between the number of body regions affected by WMSDs and QoL (*P* for linear trend test = 0.001). Moreover, participants with three or more days off per week reported significantly higher QoL scores (β = 5.64; 95% CI, 1.45 to 9.83) compared to those with only one day off per week.

However, as shown in Table 4, these associations varied by specific domains. Each increased body region affected by WMSDs was significantly associated with decreases of 0.722 (*p* = 0.001) in overall QoL, and of 0.262 (*p* < 0.001), 0.153 (*p* = 0.022), 0.148 (*p* = 0.035), and 0.159 (*p* = 0.007) in the physical, psychological, social, and environmental domains, respectively. When the number of affected regions was dichotomized at the median, participants with a median or greater number of body regions affected by WMSDs had significantly lower QoL scores in the physical domain (β = -1.17; 95% CI, -1.75 to -0.60) and environmental domain (β = -0.74; 95% CI, -1.35 to -0.14) compared to those with fewer than the median number of affected regions, while no significant differences were observed in the other domains. Additionally, compared with those who had an average of only one day off per week, participants with an average of three or more days off per week exhibited significantly higher QoL scores in the physical domain (β = 1.43; 95% CI, 0.36 to 2.50), psychological domain (β = 1.57; 95% CI, 0.29 to 2.84), and environmental domain (β = 1.73; 95% CI, 0.60 to 2.86). Participants holding the academic title of associate professor had significantly lower QoL scores in the domain of social relationships (β = -0.93; 95% CI, -1.80 to -0.06) compared to those being assistant professor.

## Discussion

### Main findings

This study provides evidence that both the burden of WMSDs and selected work-related characteristics are associated with QoL among university teachers with WMSDs. Overall, teachers with a greater number of WMSD-affected body regions tended to report poorer QoL. The findings also indicate that adequate recovery time, reflected by ≥ three days off per week, is associated with better QoL, whereas associate professors may be particularly vulnerable to poorer social QoL. Taken together, these results highlight the importance of considering both musculoskeletal health and work-related conditions when developing strategies to improve the well-being of university teachers.

### Quality of life among university teachers

Compared with the mean overall QoL reported for the general workforce in Taiwan [42], university teachers in this study exhibited a lower QoL score (57.17 ± 8.80). Furthermore, their QoL was slightly lower than that reported in studies from several other countries using the WHOQOL-BREF. For instance, a study on female university teachers in Saudi Arabia reported a higher average overall QoL score of 58.32 [25]. Similar patterns were observed across specific QoL domains. In a Saudi Arabian study, scores in the physical (15.09), social (14.61), psychological (14.31), and environmental (14.30) domains were all larger than those observed in the present study [25]. Another study conducted in Brazil among nursing faculty members found even higher scores: psychological (16.52), social (16.54), physical (15.92), and environmental (14.96) [27]. One possible explanation for the discrepant findings is that WMSD severity may have been greater among participants in our study than in prior research, as only individuals with WMSDs were included [25, 27]. Additionally, evidence from cross-national comparisons suggests that university teachers in Taiwan may experience a heavier effective workload than their counterparts in other countries [43]. Taiwanese university teachers tend to maintain teaching-related activities during academic breaks, unlike those in countries such as Japan and the United States, where teaching responsibilities are substantially reduced [43]. Moreover, research time does not increase correspondingly during these periods, indicating a constrained time-allocation structure [43]. Furthermore, a higher student–faculty ratio and the requirement to simultaneously fulfill teaching, research, and administrative responsibilities further intensify workload demands [43]. Collectively, these factors contribute to a more demanding academic work environment in Taiwan, which may lead to greater occupational stress and poorer QoL among university teachers. Interestingly, consistent with prior studies reporting reduced psychological QoL among university teachers [25, 28–30], domain-specific QoL scores in this study were 14.46 ± 2.47 (physical), 14.01 ± 2.57 (psychological), 14.11 ± 2.62 (social), and 14.59 ± 2.33 (environmental), with the psychological domain lowest. This may suggest that, irrespective of WMSDs, the psychological domain of QoL among university teachers is more severely impaired than other QoL domains across many countries [25, 28–30], including Taiwan. The QoL in psychological domain may be more severely affected by the demands and pressures of academic work. Prior research has shown that psychological and emotional symptoms are the most common stress responses among teachers [44]. Therefore, it is crucial for universities and related institutions to pay greater attention to the QoL of teachers, particularly in the psychological domain.

### Association between WMSDs and QoL

Previous studies have reported that university teachers with MSD discomfort in more than 50% of body regions exhibit lower QoL scores in the physical and mental domains, as measured by the SF-36 [10, 11] or SF-12 [13]. In contrast to these studies [10, 11, 13], our study identified a significant dose–response relationship between the number of body regions affected by WMSDs and both overall and domain-specific QoL; however, this association changed when WMSD sites were categorized using the median (50th percentile; <4 vs. ≥4 body regions). Specifically, teachers with a higher number of affected body regions (≥ median) did not show significantly lower QoL in the psychological and social domains compared with those with fewer affected regions. Nevertheless, our findings indicate that greater musculoskeletal discomfort was most strongly associated with reduced scores in the physical subdomains (pain, mobility, fatigue, sleep, and daily activities), followed by the environmental subdomains (safety and physical environment), even after adjusting for personal and work-related factors in regression analyses, highlighting its impact on physical functioning and sensitivity to environmental stressors.

The mechanisms by which factors affect the dose-response association between number of body regions affected by WMSDs and QoL among university teachers have also not been fully identified. However, previous studies have reported a strong positive association between the prevalence and severity of WMSDs across affected body regions [4]. Therefore, a greater number of WMSD sites and increased symptom severity are more likely to adversely affect the physical domain of QoL. Moreover, limitation of motion was used as an indicator of MSD severity [45]. Therefore, the significant dose–response association between the number of WMSD sites and environmental QoL may be explained by greater MSD severity, which may limit physical activity and, consequently, reduce engagement with the physical environment. Furthermore, no dose–response relationship was observed for the psychological and social QoL domains when the number of WMSD sites was analyzed as a dichotomous variable. One possible explanation may be provided by the biopsychosocial (BPS) model of pain management [46]. The BPS model posits that biomedical approaches alone are insufficient for effectively managing pain, particularly MSDs; therefore, a more holistic approach that addresses biological factors, environmental influences, and cognitive aspects of pain perception is essential [47, 48]. Based on this model, we speculate that university teachers in Taiwan may possess more adaptive illness perceptions, favorable sociocultural beliefs, and effective coping mechanisms, particularly among those experiencing a higher number of WMSD sites (≥ 50th percentile). These factors may buffer against emotional distress and social relationship impairment, thereby mitigating the adverse impact of symptoms on psychological and social domains of QoL.

### Associations between work-related factors and QoL

In addition to the number of WMSD sites, our study identified average days off per week and academic rank as significant work-related factors associated with overall and domain-specific QoL among university teachers with WMSDs. This contrasts with previous studies, which reported daily working hours [9], administrative workload [26, 28], and teaching load [28] as significant work-related factors. This discrepancy might be a result from differences in the previous studies did not include variable such as average days off per week and breaktime to rest at work into analysis [9, 26, 28]. Unlike previous studies, this study included work–rest variables, revealing that average days off per week was the strongest predictor of overall QoL. Teachers with three or more days off scored 5.64 points higher (95% CI: 1.45–9.83) than those with one day off. The positive association was significant for physical, psychological, and environmental domains, but not for social QoL. Several possible explanations may account for these findings. One possible explanation is that discomfort associated with WMSDs increases physical demand, particularly among teachers experiencing more severe symptoms due to higher WMSD prevalence, and that rest days serve as a critical recovery resource, enabling university teachers to implement coping strategies more effectively to manage these WMSD-related discomforts [4]. Another possible explanation may be that physical fatigue may mediate relations between rest time, and QoL among university teachers. Although Taiwanese labor laws specify an 8-hour workday or 40-hour workweek for university teachers [49], many faculty members work beyond these limits to conduct research [17], prepare lectures, and write academic papers [18–20]. Such extended work demands may be linked to greater physical fatigue, thereby lowering physical QoL. In addition, limited rest (e.g., only one day off per week) may be associated with accumulated fatigue, which in turn erodes mental health and overall QoL [50]. Therefore, we suspect that insufficient recovery resulting from limited rest may be associated with a hindered work-life balance and increased physical and psychological distress [51, 52], with fatigue serving as a potential mediator that could negatively impacting overall QoL. In contrast, longer rest periods were associated with higher scores in the physical and psychological domains, likely due to reduced physical fatigue. Additionally, more days off allow teachers to engage in leisure, healthcare, skill development, and other activities, enhancing environmental QoL.

Notably, our study found that rest days did not significantly influence the social domain of QoL, and no significant associations were observed between any domains of QoL and daily working hours, daily teaching hours, or administrative workload. The results may be explained by the social support theory [53], Job Demands-Resources (JD-R) model [54] or effort-recovery theory [55]. Social support theory suggests that supportive relationships with colleagues, administrators, and family can buffer teachers against work-related stress and burnout, potentially enhancing their QoL [53, 56, 57]. Given that the core of social QoL lies in the quality of interpersonal relationships, increases in rest time or reductions in teaching and administrative duties are unlikely to yield meaningful improvements in social QoL if teachers’ social networks are limited or the quality of their interactions is low. Therefore, we did not find that rest time or time spent on teaching and administrative duties was associated with social QoL. Similarly, according to the JD-R model [54], days off function as a resource that primarily facilitates physical recovery and reduces work-related exhaustion, thereby potentially enhancing the physical, psychological, and environmental domains of QoL. However, social QoL may be more strongly influenced by social support, the quality of interpersonal interactions, and opportunities for social engagement [53, 58] than by increased time off alone. Consequently, even if the number of rest days increases, social QoL may not improve significantly in the absence of resources that promote social interaction, such as family support or meaningful social opportunities. Moreover, evidence suggests that although long working hours are typically associated with high job demands and work overload, they do not necessarily lead to greater fatigue, which may be attributable to university teachers’ abundant job resources that enable effective management of their workload [54, 59]. The observation that university teachers’ substantial job resources—such as autonomy, recognition, and opportunities for professional development—may alleviate fatigue can be explained by the JD-R model [54]. Similarly, as proposed by the effort–recovery theory [55], resource-rich work environments encourage employees to invest effort willingly and recover efficiently, thereby maintaining their well-being. Taken together, these perspectives suggest that the absence of a relationship between working or teaching hours and QoL in our study may be attributed to the buffering role of job resources, which mitigate the impact of job demands on fatigue and consequently preserve university teachers’ QoL.

Interestingly, academic rank was the only work-related factor significantly associated with social QoL in our study. Associate professors exhibited significantly lower social QoL than assistant professors, whereas no significant difference was observed between full and assistant professors. This finding is inconsistent with prior evidence suggesting that, within the context of Chinese Confucianism, higher academic rank is associated with better interpersonal relationships and greater social prestige, thereby contributing to improved social QoL [9]. The underlying reasons for this pattern remain unclear; however, the association between academic rank and social QoL may be explained by role strain theory [60] and JD-R model [54]. In general, associate professors—who occupy the career stage preceding promotion to full professor—often face increased demands for institutional and interpersonal service, commonly referred to as “invisible labor” [23]. This labor encompasses efforts to develop others’ abilities, provide mentorship, maintain relationships that support colleagues and students, and engage in unseen administrative tasks such as committee work, meetings, and problem-solving within the institution [23]. Such unrecognized work frequently arises from implicit organizational expectations to serve others in ways that are neither formally rewarded nor adequately evaluated within academic promotion systems [23]. Consequently, these obligations may divert time and energy from scholarly activities critical for career advancement. To preserve research productivity, associate professors may limit opportunities for social engagement to allocate more time to research. Another possible explanation is that associate professors often receive less mentorship than during their assistant professor years [23], reflecting a reduction in available social support at this career stage. In this context, associate professors simultaneously occupy multiple roles while resources remain limited or insufficiently matched, which may constrain both the time and quality of social interactions, thereby reducing social QoL. This phenomenon can be explained by role strain theory [60], which posits that individuals occupying multiple roles may experience overload and conflict when role demands exceed available resources, ultimately undermining their social functioning. From the perspective of the JD-R model, although professors typically face higher job demands associated with research, teaching [22], and administrative responsibilities, they also have greater job resources—such as extensive social support networks [9], autonomy, recognition [24], and compensation [61]—which may buffer the impact of work-related interpersonal stress on their social QoL.

### Contributions and limitations

To our knowledge, this cross-sectional study is the first to investigate the associations between the number of body sites affected by WMSDs, work-related factors, and multiple domains of QoL—including physical, psychological, social, and environmental dimensions—among university teachers with WMSDs. Our study advances beyond the conventional approach of relying solely on median-based (50th percentile) categorization of WMSD sites [10, 11, 13] by treating the number of WMSD sites merely as a continuous variable to examine the dose–response association between WMSD burden and QoL. Additionally, our study is distinguished from previous research by incorporating often overlooked work-related factors—such as teaching hours, number of days off, and opportunities for rest breaks—into multivariable regression analyses [9, 24, 26, 28, 31, 32, 59]. Furthermore, we extend prior work that has primarily focused on overall QoL [24, 26, 28, 31, 59] or a limited set of domains (e.g., physical and mental) [9, 32] by comprehensively evaluating these associations across overall QoL and its physical, psychological, social, and environmental dimensions.

However, its cross-sectional design limits causal inference. Second, participants recruitment might have introduced self-selection bias due to the convenience sampling, leading to the concern of representativeness of the sample. Thus, the findings should be interpreted with caution and may primarily apply to volunteer university teachers with characteristics similar to those in our sample rather than to the entire population of Taiwanese university faculty. Third, certain factors that may influence QoL, such as organizational behavior-related variables [32], and fatigue [50] were not included in the analysis. Future research should consider incorporating these factors to provide a more comprehensive understanding of QoL among university teachers with WMSDs. For instance, further investigation is warranted to elucidate the mechanisms linking limited rest (e.g., only one day off per week) to poorer psychological QoL, with particular attention to the potential mediating role of fatigue in longitudinal or mediation studies. Finally, reliance on self-reported WMSDs and QoL may introduce recall bias, warranting cautious interpretation of the findings.

## Conclusions

University teachers with WMSDs reported relatively low QoL, particularly in the psychological domain. A greater number of body regions affected by WMSDs was associated with poorer QoL, while having three or more days off per week was associated with better QoL. In addition, academic rank was related to social relationship domain of QoL, with associate professors showing lower score than assistant professors. These findings suggest that improving musculoskeletal health and ensuring adequate opportunities for recovery may contribute to better QoL among university teachers. Universities should consider implementing occupational health interventions, promoting sufficient rest, and strengthening psychosocial support, particularly for teachers experiencing a high burden of WMSDs and for associate professors.

## Data Availability

The datasets generated and/or analyzed during the current study are not publicly available to ensure data confidentiality, as specified in the consent form and in accordance with the ethics approval, to prevent the disclosure of identifying information that could compromise participant privacy. However, they are available in an anonymized form from the corresponding author on reasonable request.
